# CRISPR/Cas9-mediated genome editing in vancomycin-producing strain *Amycolatopsis keratiniphila*

**DOI:** 10.3389/fbioe.2023.1141176

**Published:** 2023-03-03

**Authors:** Mengyi Hu, Shuo Chen, Yao Ni, Wei Wei, Wenwei Mao, Mei Ge, Xiuping Qian

**Affiliations:** ^1^ School of Pharmacy, Shanghai Jiao Tong University, Shanghai, China; ^2^ Shanghai Laiyi Center for Biopharmaceutical R&D, Shanghai, China

**Keywords:** amycolatopsis, CRISPR/Cas9, genome editing, large fragment deletion, Eco-0501, vancomycin

## Abstract

*Amycolatopsis* is an important source of diverse valuable bioactive natural products. The CRISPR/Cas-mediated gene editing tool has been established in some *Amycolatopsis* species and has accomplished the deletion of single gene or two genes. The goal of this study was to develop a high-efficient CRISPR/Cas9-mediated genome editing system in vancomycin-producing strain *A. keratiniphila* HCCB10007 and enhance the production of vancomycin by deleting the large fragments of ECO-0501 BGC. By adopting the promoters of *gapdh*p and ermE*p which drove the expressions of *scocas9* and sgRNA, respectively, the all-in-one editing plasmid by homology-directed repair (HDR) precisely deleted the single gene *gtfD* and inserted the gene *eGFP* with the efficiency of 100%. Furthermore, The CRISPR/Cas9-mediated editing system successfully deleted the large fragments of *cds13-17* (7.7 kb), *cds23* (12.7 kb) and *cds22-23* (21.2 kb) in ECO-0501 biosynthetic gene cluster (BGC) with high efficiencies of 81%–97% by selecting the sgRNAs with a suitable PAM sequence. Finally, a larger fragment of *cds4-27* (87.5 kb) in ECO-0501 BGC was deleted by a dual-sgRNA strategy. The deletion of the ECO-0501 BGCs revealed a noticeable improvement of vancomycin production, and the mutants, which were deleted the ECO-0501 BGCs of *cds13-17*, *cds22-23* and *cds4-27*, all achieved a 30%–40% increase in vancomycin yield*.* Therefore, the successful construction of the CRISPR/Cas9-mediated genome editing system and its application in large fragment deletion in *A. keratiniphila* HCCB10007 might provide a powerful tool for other *Amycolatopsis* species.

## 1 Introduction

*Amycolatopsis*, a crucial genus of actinomycetes, was firstly defined as a new genus in 1986 with the feature of type IV cell wall composition and lacking mycolic acids (Lechevalier et al., 1986), and 86 species with validly published names have been described as of 2022 (https://lpsn.dsmz.de/genus/amycolatopsis). The genus *Amycolatopsis* is regarded as an important source for the generation of diverse valuable bioactive secondary metabolites (Song et al., 2021), such as antibiotics [vancomycin (Barna and Williams, 1984), rifamycin (Saxena et al., 2014), chloroeremomycin (Lu et al., 2004), balhimycin (Frasch et al., 2015), and ECO-0501 (Banskota et al., 2006)]. Several *Amycolatopsis* species were applied in bioremediation (heavy metal immobilization, herbicide and polymer biodegradation) and bioconversion (wuxistatin and vanillin production) (Kisil et al., 2021).

The genome sequence analysis of *Amycolatopsis* spp*.* (http://wwws.ncbi.nlm.nih.gov/genomes/lproks.cgi) has revealed that the strains of *Amycolatopsis*, which have comparatively large genomes (from 5 to 10 Mb) in the form of a circular chromosomes, contain over 20 BGCs of natural products and the majority of BGCs are rarely or even not expressed under typical laboratory culture conditions (Kumari et al., 2016). The efficient genome editing tools not only can discover new valuable compounds by activating silent BGCs (Choi et al., 2015; Katz and Baltz, 2016; Nguyen et al., 2020), but also can improve the yield and purity of target metabolites and enhance the strain stability by amplifying BGC copy numbers, deleting genes for competing pathways, manipulating positive and negative regulatory genes, expressing BGCs in heterologous hosts, or refactoring the transcription and translation process, and so on (Baltz, 2016; Horbal et al., 2018). Among actinomycetes, CRISPR/Cas-based genetic engineering has been the most extensively investigated and widely applicated system in *Streptomyces* species, and it accelerated the natural product discovery, strain improvement, and functional genome research by single or multiplex gene/genome editing with higher efficiencies (Cobb et al., 2015; Tong et al., 2015; Wang et al., 2016; Tao et al., 2018; Alberti and Corre, 2019). The genetic manipulation in *Amycolatopsis* has progressed slowly due to the scarcity of sophisticated genetic tools and methods, such as strain-compatible tools, cloning methods for high GC-content DNA sequence, and transfer methods (Malhotra and Lal, 2007; Meyer et al., 2017; Mitousis et al., 2020). At present, the highly efficient CRISPR/Cas12a-based genome editing systems were developed in *A. mediterranei* U32 and *A. orientalis* AO-1, and deleted *rifZ*, *glnR*, and *gtfDE* genes successfully (Zhou et al., 2020; Qian et al., 2021). Furthermore, a CRISPR/Cas9 system could delete *vdh* gene with the efficiency of 10% in *Amycolatopsis* sp. (Zheng et al., 2021). However, these studies only conducted the knock-out of small fragments of about 1–3 kb. It is still crucial and challenging to develop a highly efficient CRISPR/Cas system for manipulating large DNA fragments in *Amycolatopsis*. The industrial strain of *A. keratiniphila* HCCB10007 has been used for large-scale production of the vital antibiotic vancomycin, and 26 gene clusters related to secondary metabolism were identified in the genome (Xu et al., 2014). The glycosidic polyketide antibiotic of ECO-0501, which discovered from the vancomycin-producer strain by genome scanning, was another important bioactive secondary metabolite (Banskota et al., 2006; Shen et al., 2014). The aim of this study was to develop a highly efficient CRISPR/Cas9-mediated editing system for deleting large fragments of ECO-0501 BGC in *A. keratiniphila* HCCB10007 and improve the production of vancomycin.

The all-in-one single plasmid pKCcas9dO consists of a target-specific guide RNA (sgRNA), a codon-optimized *cas9* (*scocas9*), two HDR templates and the temperature-sensitive replicon pSG5, and the system was employed by Huang et al., 2015 to create single/double gene deletions, single/double large-size gene cluster deletions, and point mutations in *S. coelicolor* with high efficiencies. The homologous regions flanking the editing sites in the system were used as a template for DNA double-strand breaks (DSBs) recombination repair (Huang et al., 2015), and provided more efficient and accurate target gene editing (Tong et al., 2015; Wang et al., 2016; Zhang et al., 2020; Zhou et al., 2020).

Here, the CRISPR/Cas9-mediated genome editing system derived from the plasmid pKCcas9dO was established in *A. keratiniphila* HCCB10007. The CRISPR/Cas9-mediated editing system could delete and insert single gene precisely, accomplish a high-efficient deletion of large-size DNA fragments of 21 kb by choosing proper sgRNA, and achieve a larger DNA fragment of 87.5 kb deletion by dual-sgRNA-guided cleavage strategy. It drastically improved the genome editing efficiency in *A. keratiniphila* and increased the production of vancomycin by deleting the competing biosynthetic pathway of ECO-0501.

## 2 Materials and methods

### 2.1 Strains, plasmids, and cultivation conditions

The strains and plasmids used in this study are listed in Supplementary Table S1. The cells of *E. coli* DH5α and *E. coli* JM110 were grown in Luria−Bertani medium at 37°C for 12–16 h. All *A. keratiniphila* strains were grown at 28°C. For sporulation, the cells were grown on Gauze’s synthetic agar medium (GM). For preparing the competent cells, *A. keratiniphila* HCCB10007 was cultured in complete pre-cultivation medium (CRM) for 48 h. For the selection of transformants, the cells of *A. keratiniphila* were grown on Bennet’s medium for 4 days, and then were cultivated in tryptic soy broth (TSB) liquid medium for 2 days. All of the above strains were cultured with agitation at 220 rpm in liquid medium. When necessary, 50 μg/mL or 100 μg/mL of apramycin (Apr) was added in the liquid medium or solid medium. For vancomycin and ECO-0501 production, *A. keratiniphila* HCCB10007 and the mutants were cultivated in seed medium for about 60 h with shaking at 250 rpm, and were then incubated at 200 rpm for 4 days (Shen et al., 2014; Xu et al., 2014).

### 2.2 Primers and reagents

The primers used in this study are listed in Supplementary Table S2. Restriction enzymes, Taq enzymes and ligases, and other common molecular biology reagents were purchased from TaKaRa. The ClonExpress MultiS One Step Cloning Kit purchased from Vazyme Biotech Co., Ltd. was used for ligation of fragments and vectors by homologous recombination. High fidelity polymerase KOD FX and PrimeSTAR (Toyobo) were used to amplify target gene for cloning purposes and to perform PCR screening of mutant strains according to the manufacturer’s protocol. PCR reactions were carried out in a PCR instrument (Eppendorf). The sequencing of DNA and the synthesis of all primers were conducted by GENEWIZ. DNA recovery kits and plasmid extraction kits were purchased from Toyobo, and a DNA Marker (GenerulerTM 1 kb DNA ladder) was purchased from Fermentas. All chemicals used were analytical grade and commercially available.

### 2.3 DNA manipulations

Isolation of genomic DNA from *Amycolatopsis* strains and plasmid DNA from *E. coli* were carried out using standard protocols (Kieser et al., 2000). Restriction enzymes and molecular biology reagents were used according to recommendation of suppliers (Takara, Vazyme, Toyobo, Eppendorf).

### 2.4 sgRNA design

The CRISPR/Cas9 target online predictor CCTop (http://crispr.cos.uni-heidelberg.de), which present the rapid selection of high quality target sites for NHEJ as well as HDR, was selected for the guide sequences and protospacer adjacent motif (PAM) of sgRNA design (Stemmer et al., 2015; Labuhn et al., 2018).

### 2.5 Construction of CRISPR/Cas9 editing plasmids for *gtfD* deletion and *eGFP* insertion

On the basis of pKCcas9dO (Huang et al., 2015), the plasmids containing different promoters to drive the expression of *cas9* and sgRNA were constructed as follows. 1) Using the plasmid pKCcas9dO as the template, the sgRNA fragment containing the promoter *J23119*, the crRNA scaffold and 19-nt direct repeat was amplified with the primers gRNADNrecom/gtfDgRNAspc2. The resulting sgRNA fragment was cloned into the *Spe*I/*Hin*dIII-digested pKCcas9dO by the Solution-I ligation and the plasmid pKCcas9dgtfD-NA was generated. 2) The sgRNA fragment containing the crRNA scaffold and 19-nt direct repeat was amplified from pKCcas9dO with the primers gRNADNrecom/gtfDgRNArecom. The ermE* promoter was amplified from pLYZWG using the primers ermE-F/ermE-R (Xu et al., 2015). With the Gibson method, the resulting sgRNA fragment and the ermE* promoter was recombined into the plasmid pKCcas9dO which was doubly digested by *Xba*I/*Hin*dIII, and the plasmid pKCcas9EgdgtfD-NA was generated. 3) The endogenous *gapdh* promoter was amplified from the genome of *A. keratiniphila* HCCB10007 using the primers gapdh-F/gapdh-R, and it was cloned into the *Xba*I/*Nde*I-digested pKCcas9EgdgtfD-NA by Gibson ligation, thus generating the plasmid pKCpGcas9EgdgtfD-NA.

**FIGURE 1:**
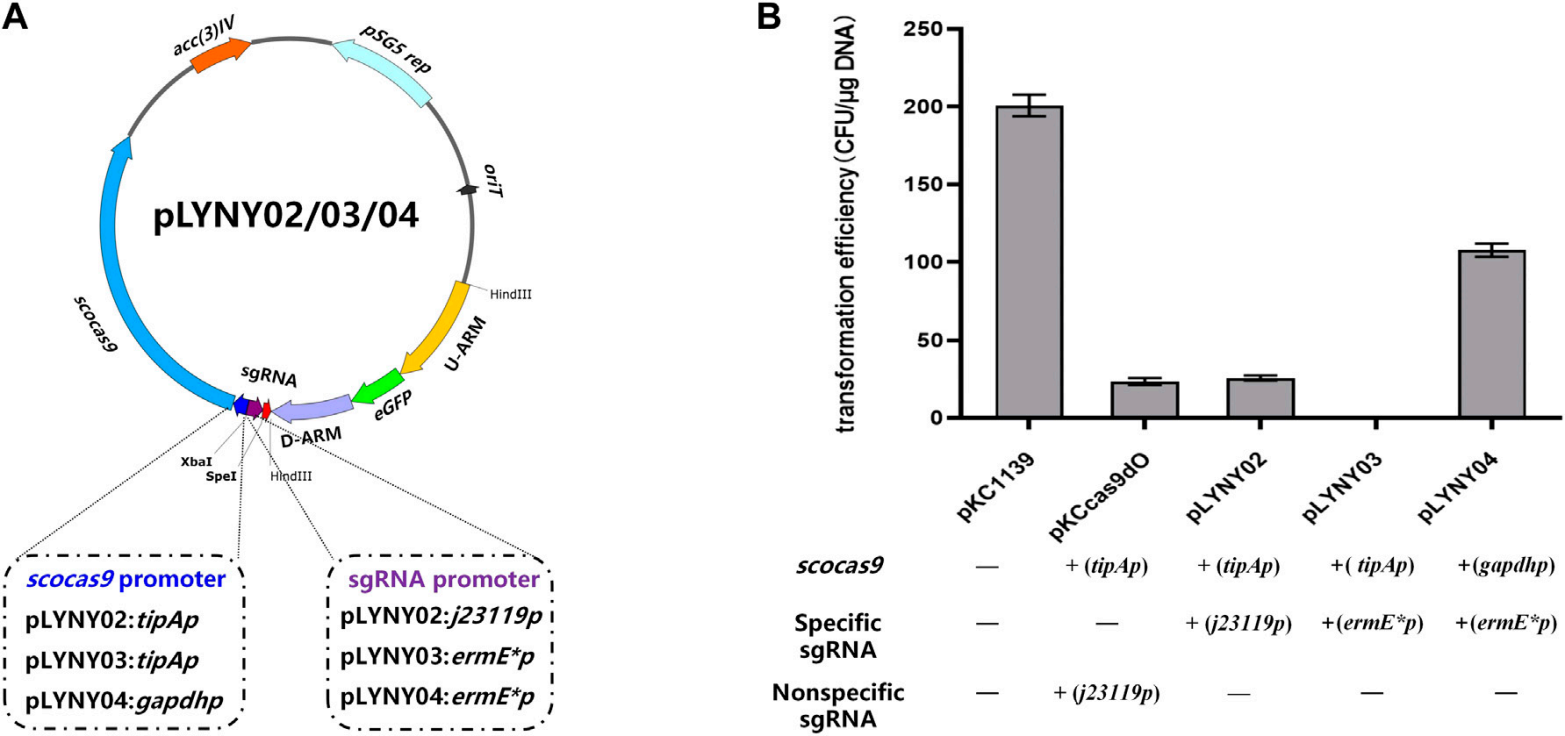
The CRISPR/Cas9-mediated editing plasmids for targeted gene editing in *A. keratiniphila*. **(A)** Schematic diagram of the editing plasmids pLYNY02, pLYNY03, and pLYNY04. Notable features included a codon-optimized *scocas9* driven by *tipA* promoter or *gapdh* promoter, target-specific sgRNA expression cassette driven by *j23119* promoter or ermE* promoter, the homologous arms flanking the target gene, a temperature-sensitive pSG5 origin, and selection marker *aac(3)IV*. **(B)** Effects of different CRISPR/Cas9-mediated editing plasmids on the transformation efficiency. The high expression of *scocas9* under control of the inducible promoter *tipA* had toxicity and prevented the cell growth. The number of transformants was greatly decreased with pLYNY02, where no transformants were obtained with pLYNY03. When *scocas9* was driven by the endogenous promoter *gapdh* from *A. keratiniphila* HCCB10007, the transformation efficiency was significantly improved. The plasmids pKC1139 and pKCcas9dO were used as the controls. Error bars represented the standard deviations from three independent biological replicates.

Using the genomic DNA of *A. keratiniphila* HCCB10007 as the template, the upstream and downstream homologous arm fragments of the gene *gtfD* were obtained by PCR amplification using the primers Vcm-8F/Vcm-8R and Vcm-10F/Vcm-10R. The two fragments were recovered with a DNA recovery kit and then fused into the pMD19-Tsimple vector by overlapping extension PCR. The resulting plasmid was doubly digested by *KpnI/PstI* and ligated with *eGFP* fragment, which was originated from pLYZWG by double digestion with *Kpn*I/*Pst*I (Xu et al., 2015). The fragment of *eGFP* was inserted between the upstream and downstream homologous arms of *gtfD* by the Solution-I ligation. The homologous arms inserted by *eGFP* were integrated into pKCcas9dgtfD-NA, pKCcas9EgdgtfD-NA, and pKCpGCas9EgdgtfD-NA by using a homologous recombination kit to generate the CRISPR/Cas9 editing plasmids pLYNY02, pLYNY03, and pLYNY04. The *cas9* was driven by *tipA* or *gadph* promoter and the target-specific sgRNA was driven by *J23119* or ermE* promoter, respectively. Figure 1A showed the detailed CRISPR/Cas9 editing plasmids pLYNY02, pLYNY03, and pLYNY04.

### 2.6 Construction of CRISPR/Cas9 editing plasmids for deleting the fragments of ECO-0501

Based on pLYNY04, a series of CRISPR/Cas9-mediated editing plasmids containing different target-specific sgRNAs and homologous DNA arms for deleting the fragments of *cds13-17* (7.7 kb), *cds23* (12.7 kb), *cds22-23* (21.2 kb), and *cds4-27* (87.5 kb) in ECO-0501 BGC were constructed, respectively. Taking the editing plasmid for knocking out *cds13-17* as an example, the upstream and downstream homologous regions of *cds13-17* were amplified from the genome of *A. keratiniphila* HCCB10007 using the primers 13-17-arm-AF/13-17-arm-AR and 13-17-arm-ZF/13-17-arm-ZR, respectively. These two fragments were recombined with the linear pLYNY04 vector generated by *Hind*III digestion. This resulting plasmid was linearized by *Spe*I digestion and ligated with annealed sgRNA oligonucleotide by overlapping recombination to obtain the final *cds13-17*-specific editing plasmid.

The dual sgRNA-guided plasmid pLYHMY87-5-I was constructed as follows. The plasmid pLYHMY7-5 was amplified with the primers of sgRNA5-F/sgRNA5-R, and the fragment containing sgRNA 5 and *ermE* ^*^ promoter was purled and ligated into pLYHMY21-I digested by *Xba*I.

### 2.7 Construction of the *A. keratiniphila* HCCB10007 mutants

The plasmids were transferred into *E. coli* DH5α for cloning and *E. coli* JM110 for demethylation by heat shock following the manufacturer’s suggested protocol. The competent cells of *A. keratiniphila* HCCB10007 for electroporation were prepared as previously described (Xu et al., 2015). The CRISPR/Cas9 editing plasmids were transformed into the competent cells of *A. keratiniphila* HCCB10007 by a Gene Pulser Xcell (Bio-Rad, Inc.) and electroporation was performed at a field strength of 7.5 kV/cm (25 μF, 600 Ω) with a pulse of about 13 ms. After electroporation, 1 mL of liquid TSB was added to the cell suspension, followed by 7 h incubation. The transformants were grown on Bennet’s medium containing Apr for 5 days, and the incubation was continued in 3 mL of liquid TSB medium containing Apr at 200 rpm for 2 days. The resulting colonies were subsequently checked by PCR with a set of primers outside or inside the region of recombination and subsequent Sanger sequencing. These verified colonies were cultivated on Bennet’s medium at 37°C overnight for one or two rounds to eliminate the plasmid, and subsequently incubated on Bennet’s medium with or without Apr for 3 days at 28°C to select the correct mutants.

### 2.8 Analyses of vancomycin and ECO-0501

The analyses of vancomycin and ECO-0501 produced by the strains of *A. keratiniphila* HCCB10007 and the mutants were conducted as previously described (Shen et al., 2014; Xu et al., 2014).

### 2.9 Fluorescence microscopy

The strains of *A. keratiniphila* HCCB10007 and *A. keratiniphila* HCCB10007 *eGFPΔgtfD* were cultured in TSB medium for 5 days. The mycelia were observed under a Leica TCS-SP8 confocal laser-scanning microscope with a ×40 objective lens using excitation wavelength (485 nm) and emitting wavelength (535 nm) (Santos-Beneit and Errington, 2017).

## 3 Results

### 3.1 CRISPR/Cas9-mediated deletion and insertion of single gene

**FIGURE 2:**
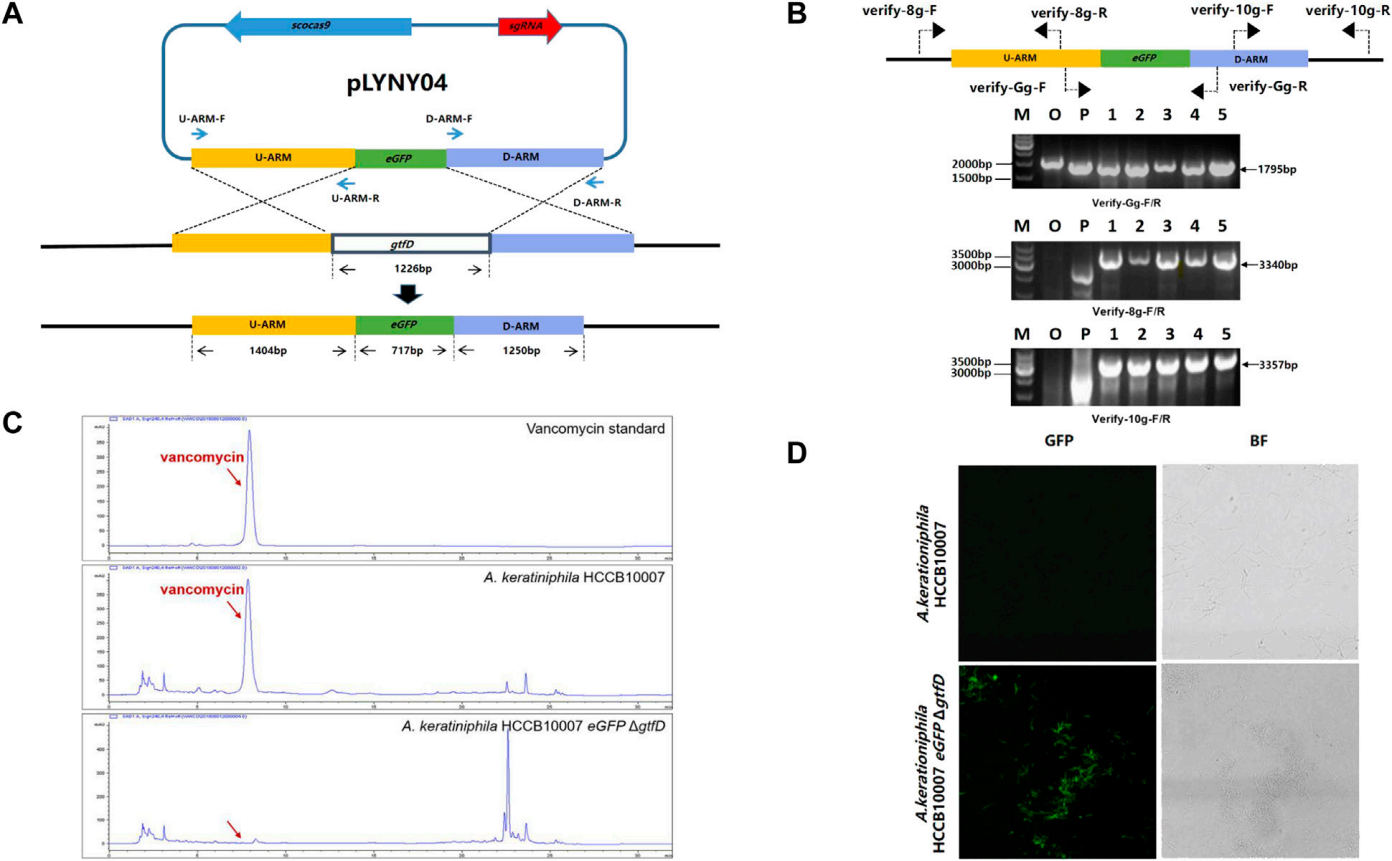
CRISPR/Cas9-mediated the deletion of *gtfD* and the insertion of *eGFP* in *A. keratiniphila.* **(A)** Strategy for replacement of *gtfD* by *eGFP* with CRISPR/Cas9-mediated editing plasmid pLYNY04. Scocas9 introduced DSB within *gtfD* gene, which could then be precisely repaired by a donor DNA fragment containing *eGFP* and the flanking upstream and downstream homologous arms. **(B)** PCR amplification analysis of randomly selected transformants verified the simultaneous *gtfD* deletion and *eGFP* insertion. The 1795 bp amplicons represented the replacement of *gtfD* by *eGFP*, and the 3340bp and 3357bp PCR amplicons verified the insertion of *eGFP*. M, GeneRuler 1 kb DNA Ladder (Thermo Scientific). O, original strain *A. keratiniphila* HCCB10007. P, pLYNY04. Lanes 1–5, colonies transformed with pLYNY04. **(C)** HPLC analysis of vancomycin from the original strain *A. keratiniphila* HCCB10007 and the mutant *A. keratiniphila* HCCB10007 *eGFP*Δ*gtfD.* The *gtfD* deleted mutant did not produce vancomycin. The position indicated by the red arrow was vancomycin peak. **(D)** Mycelia observation of the strain *A. keratiniphila* HCCB10007 and the mutant *A. keratiniphila* HCCB10007 *eGFP*Δ*gtfD* by confocal laser-scanning microscopy. The fluorescence of the mutant mycelia confirmed the expression of *eGFP*.

The CRISPR/Cas9-mediated editing plasmid in this study was derived from the high-efficiency plasmid pKCcas9dO, which originated from pKC1139 and successfully applied in *Streptomyces* (Huang et al., 2015). However, the transformation efficiency was much lower than that of the control plasmid pKC1139 in *A. keratiniphila* HCCB10007 (Figure 1B). To drive the expression of the *cas9* gene and the target-specific sgRNA in *A. keratiniphila*, the three editing plasmids, which *cas9* and sgRNA were expressed under control of different promoters, were transformed into *A. keratiniphila* HCCB10007 by electroporation. Compared with the empty vector pKC1139, the introduction of pKCcas9dO and pLYNY02 caused about 88% decrease in the transformation efficiency, and no transformants were observed in three independent experiments with pLYNY03. It was indicated that Cas9 toxicity was apparent for *A. keratiniphila*. When *cas9* was expressed under control of endogenous *gapdh* promoter from *A. keratiniphila* HCCB10007, the transformation efficiency was improved. About 46.5% decrease of transformation efficiency was caused when pLYNY04 was introduced into *A. keratiniphila* HCCB10007. The number of transformants was acceptable for the genetic manipulation. The deletion of glycosyltransferase gene *gtfD* and insertion of *eGFP* with the editing plasmid pLYNY04 was illustrated in Figure 2A.

After eliminating the plasmid of pLYNY04 by high-temperature culturing, the colonies were confirmed by PCR with three pairs of primers (Figure 2B). Because of the replacement of *gtfD* by *eGFP*, PCR amplification from the genomic DNA of the original strain produced no specific products, and PCR of the mutants had the amplicons of 1795, 3340, and 3357 bp, respectively. It was confirmed that the introduction of pLYNY04 into *A. keratiniphila* HCCB10007 caused a 100% editing efficiency of *gtfD* gene deletion and *eGFP* gene insertion from three independent replicates. The mutant strain was named *A. keratiniphila* HCCB1007 *eGFP* Δ*gtfD*.

As expected, the HPLC assay for the fermentation products showed that unlike the *A. keratiniphila* HCCB10007, no vancomycin peak was observed in the mutant of *A. keratiniphila* HCCB1007 *eGFP* Δ*gtfD* (Figure 2C). The absence of vancomycin in the metabolites of the mutant strain proved that the pLYNY04 plasmid successfully deleted the *gtfD* and further led to the block of vancomycin biosynthesis. The green fluorescence was detected clearly in the mycelia of *A. keratiniphila* HCCB1007 *eGFP* Δ*gtfD* by confocal laser microscopy, which further confirmed the completion of expected insertion of *eGFP* and the successful expression of green fluorescent protein (Figure 2D). It could be therefore concluded that the CRISPR/Cas9-mediated editing plasmid pLYNY04, harboring *cas9* driven by *gadph* promoter and the target-specific sgRNA driven by ermE* promoter, was capable of HDR-dependent gene editing in *A. keratiniphila* HCCB1007 and the system could achieve high efficiency for the deletion and insertion of single gene.

### 3.2 CRISPR/Cas9-mediated deletion of large fragments in ECO-0501 BGC with single sgRNA

**FIGURE 3:**
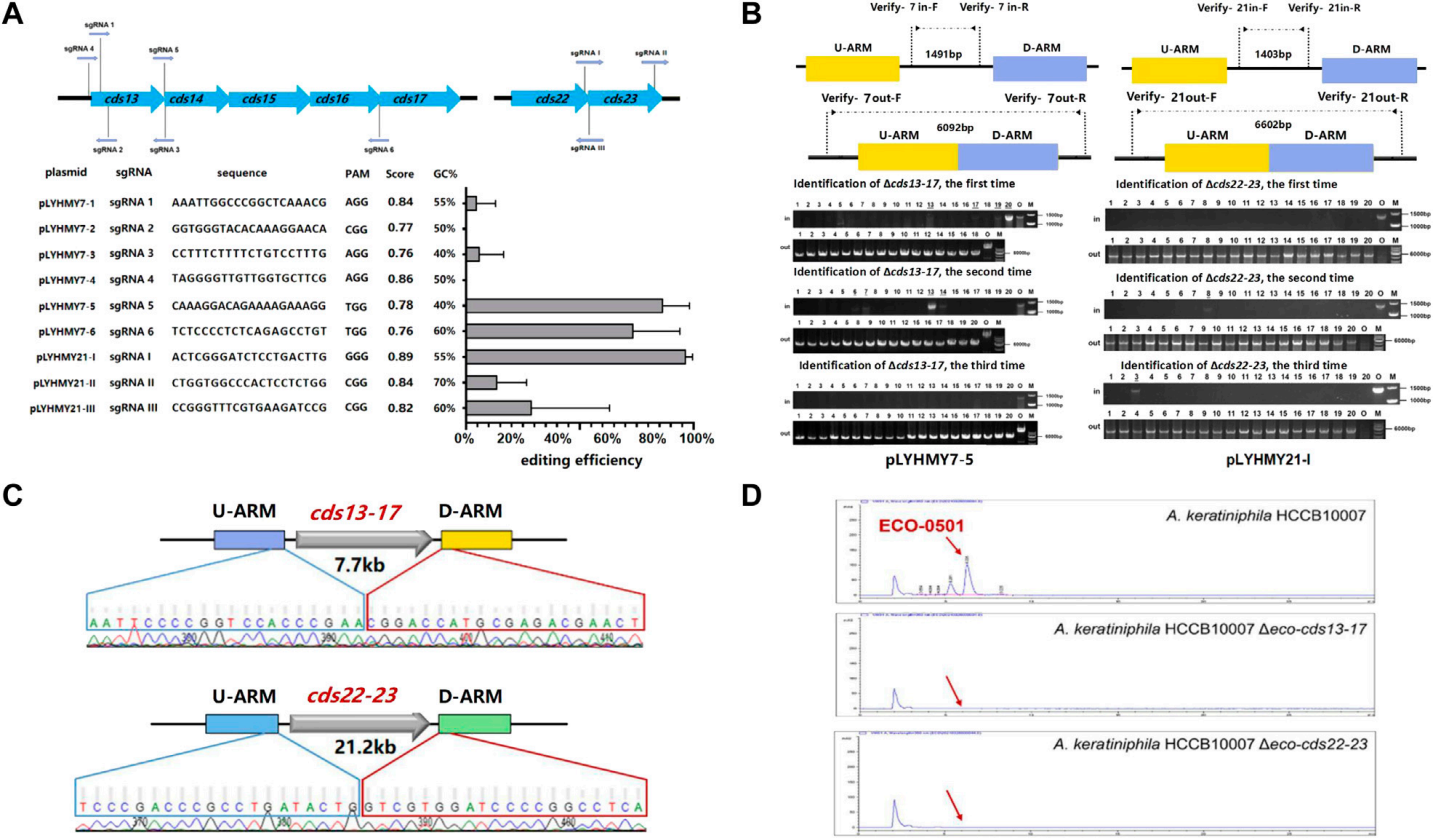
CRISPR/Cas9-mediated deletions of large fragments in ECO-0501 BGC. **(A)** The deletion efficiencies of *cds13-17* and *cds22-23* obtained by the corresponding editing plasmids. The editing plasmids contained different sgRNAs expression cassettes and up/downstream homologous arms of target clusters. Error bars represented the standard deviations from three independent biological replicates. The information of sgRNAs included the binding position, sequence composition, PAM sequence, score and GC contents. **(B)** PCR analysis of randomly selected colonies verified the deletions of *cds13-17* and *cds22-23*. The amplicons of 1491 bp and 1403 bp were the products of the original strain, respectively, and 6092 bp and 6602 bp amplicons represented that clusters *cds13-17* and *cds22-23* of colonies were deleted, respectively. The three repeated experiments of pLYHMY7-5 and pLYHMY21-I listed as representative. The underline below the number indicated that the DNA fragments of the colonies had not been deleted after the verification of Verify-7in-F/Verify-7in-R and Verify-21in-F/Verify-21in-R. M, GeneRuler 1 kb DNA Ladder (Thermo Scientific). O, original strain *A. keratiniphila* HCCB10007. Lanes 1–20, 20 colonies transformed by pLYHMY7-5 with *cds13-17*-specific sgRNA5 and pLYHMY21-I with *cds22-23*-specific sgRNAI. **(C)** Sanger sequencing results confirmed the correct deletion of the *cds13-17* and *cds22-23* clusters. **(D)** HPLC detection of ECO-0501 of the secondary metabolites in the mutants indicated that there were no ECO-0501 production owing to the successful deletion of the *cds13-17* and *cds22-23* clusters. The position indicated by the red arrow was ECO-0501 peak.

The editing ability of CRISPR/Cas9 is mainly determined by the capacity of sgRNA to recognize and cleave at specific sites, and a great quantity of online sgRNA design websites are available to predict and improve the efficiency of gene editing (Cui et al., 2018; Liu et al., 2020). The sgRNA score of CCTop is based on the off-target quality and the distribution of mismatches, and guides the user towards selecting the optimal target site (Stemmer et al., 2015). To achieve the deletion of large chromosomal fragments in *A. keratiniphila* HCCB1007, *cds13-17* (7.7 kb) and *cds22-23* (21.2 kb) in the ECO-0501 BGC were selected as targeting clusters, and nine sgRNAs targeting specific sites of *cds13-17* and *cds22-23*, which scores were over 0.75 and there were no predicted off-target effects were designed. The detailed information of location, sequence composition, PAM sequence, scores in CCTop and GC contents were shown in Figure 3A. On the basis of pLYNY04, the plasmids harboring the corresponding sgRNAs and the homologous arms of *cds13-17* or *cds22-23* were constructed, respectively. No successful deletion of *cds13-17* was observed using the plasmids of pLYHMY7-2 and pLYHMY7-4. With the plasmids of pLYHMY7-1, pLYHMY7-3, pLYHMY21-II, and pLYHMY21-III, the editing efficiencies of 5% to 29% were relatively low. The plasmids of pLYHMY7-5, pLYHMY7-6, and pLYHMY21-I exhibited the more efficient editing ability, and the editing efficiencies of 87% ± 12%, 74% ± 20%, and 97% ± 3% were accomplished, respectively for the deletion of *cds13-17* and *cds22-23* clusters (Figure 3A). The correct deletions of the clusters of *cds13-17* or *cds22-23* were verified by PCR with two pairs of primers located inside and outside of the targeting deletion clusters (Figure 3B). PCR of the *cds13-17* deleted colonies could not produce a 1491bp DNA fragment with the primers of Verify-7in-F/Verify-7in-R, while amplified a 6092bp DNA fragment with the primers of Verify-7out-F/Verify-7out-R. Similarly, the results showed that the *cds22-23* deleted colonies gave a specific 6602bp amplicon with the primers Verify-21out-F/Verify-21out-R, and did not have 1403bp amplicon with the primers Verify-21in-F/Verify-21in-R. The sequencing results of the DNA fragments deleted colonies also confirmed that the deletion of the DNA fragments of *cds13-17* or *cds22-23* and homologous recombination repair were achieved (Figure 3C). The HPLC analyses of the metabolites fermented by the two clusters deleted mutants *A. keratiniphila* HCCB10007 Δ*eco-cds13-17* and *A. keratiniphila* HCCB10007 Δ*eco-cds22-23* demonstrated that the mutant strains were unable to biosynthesize ECO-0501 and the genomic targets had been deleted correctly (Figure 3D).

**FIGURE 4:**
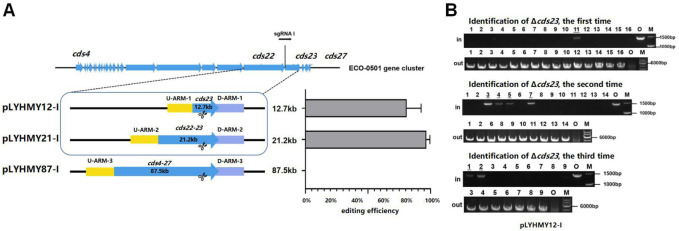
The deleting efficiencies of the same sgRNA expression cassette on different size of fragments. **(A)** The deleting efficiencies of *cds23, cds22-23*, and *cds4-27* obtained by the editing plasmids containing the same sgRNA1 expression cassette and different up/downstream homologous arms of target clusters. Error bars represented the standard deviations from three independent biological replicates. **(B)** PCR analysis of randomly selected colonies transformed with pLYHMY12-I verified the deletions of *cds23*. The amplicons of 1481 bp were the product of the original strain and 5169 bp amplicons represented that cluster *cds23* of colonies were deleted. The underline below the number indicated that the DNA fragments of the colonies had not been knocked out after the verification of Verify-12in-F/Verify-12in-R. M, GeneRuler 1 kb DNA Ladder (Thermo Scientific). O, original strain *A. keratiniphila* HCCB10007. Lanes 1–16, colonies transformed by pLYHMY12-I with *cds23-*specific sgRNA1.

To detect the editing efficiency of the same sgRNA expression cassette with the corresponding homologous arms, the plasmids of pLYHMY12-I targeting to *cds23* (12.7 kb) and pLYHMY87-I targeting to *cds4-27* (87.5 kb) were transformed into *A. keratiniphila* HCCB10007. The deletion efficiency of *cds23* was 81% ± 12%, which was comparable to the editing efficiency of 97% ± 3% which achieved by the plasmid of pLYHMY21-I for deleting *cds22-23* (21.2 kb) (Figure 4A). The correct deletion of *cds23* was validated by PCR with the corresponding verification primers. The *cds23* deleted colonies could amplify a 5169 bp fragment with the primer of Verify-12out-F/Verify-12out-R and did not obtain the amplicon of 1481 bp with primer Verify-12in-F/Verify-12in-R (Figure 4B). No mutant with deletion of *cds4-27* was obtained (Figure 4A). The plasmid of pLYHMY87-I failed to delete the fragment of 87.5 kb, which was likely due to the inefficient of single sgRNA targeting to much larger gene cluster. So, the plasmid pLYNY04 derivatives with single sgRNA and two homologous arms flanking the targeted clusters could delete large DNA fragment, which covers a size of 21.2 kb region in ECO-0501 BGC with a higher efficiency.

### 3.3 CRISPR/Cas9-mediated deletion of large fragment in ECO-0501 BGC with dual sgRNAs

**FIGURE 5:**
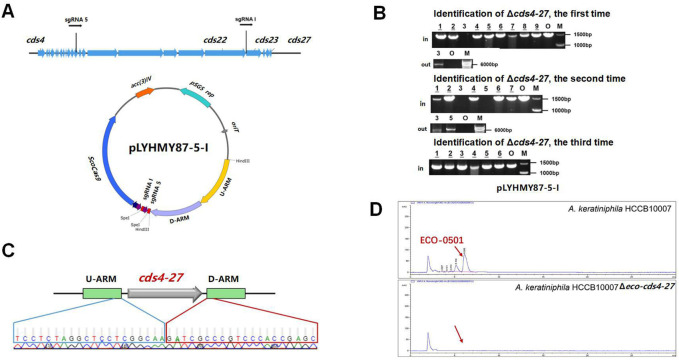
CRISPR/Cas9-mediated deletion of large fragment in ECO-0501 BGC with dual sgRNAs expression cassettes. **(A)** The location of sgRNA5 and sgRNAI on the ECO-0501 cluster and the diagram of editing plasmid PLYHMY87-5-I with dual sgRNAs expression cassette. **(B)** PCR analysis of randomly selected colonies transformed with pLYHMY87-5-I verified the deletions of *cds4-27*. The amplicons of 1403 bp were the products of the original strain and 6777 bp amplicons represented *cds4-27* deleted mutants. M, GeneRuler 1 kb DNA Ladder (Fermentas). O, original strain *A. keratiniphila* HCCB10007. Lanes 1–9, colonies transformed with pLYHMY87-5-I targeting CDS4-27. The underline below the number indicated that the gene of the colony had not been knocked out after the verification of Verify-87in-F/R. **(C)** Sanger sequencing results confirmed the correct deletion of the *cds4-27* cluster. **(D)** HPLC detection of ECO-0501 of the secondary metabolites in the mutants indicated that there were no ECO-0501 production owing to the successful deletion of the *cds4-27* cluster. The position indicated by the red arrow was ECO-0501 peak.

The dual-sgRNA strategy which introduced DSBs at both ends and bridged the gap with homologous arms clearly demonstrated that it could significantly improve the deletion efficiency of CRISPR/Cas9 system in editing larger fragments (Cobb et al., 2015; Huang et al., 2015). ECO-0501 BGC spans approximately 100 kb of DNA in *A. keratiniphila* HCCB10007 (Xu et al., 2014), The deletion of larger DNA fragments of ECO-0501 BGC is particularly valuable for increasing the availability of the precursors beneficial for vancomycin biosynthesis. To further delete the cluster of *cds4-27*, which covers 87.5 kb of DNA fragments in ECO-0501 BGC, the tandem *cds4-27*-specific sgRNA expression cassettes driven by two copies of the promoters ermE* to increase the cleavage was considered. The plasmid pLYHMY87-5-I, containing the efficient sgRNAs of both sgRNA 5 and sgRNA I with corresponding homologous arms for *cds4-27* (Figure 5A), was constructed and transformed into the strain of *A. keratiniphila* HCCB10007. The average deletion efficiency of *cds4-27* cluster was 13% ± 11%, and the cluster deleted colonies were validated by PCR with the corresponding verification primers. There was no amplicons of 1403 bp with the primer of Verify-21in-F/Verify-21in-R, and the amplicon of 6777 bp was amplified with the primer of Verify-87out-F/Verify-87out-R (Figure 5B). The DNA sequencing result of the strain *A. keratiniphila* HCCB10007 Δ*eco-cds4-27* demonstrated that the mutant deleted the cluster of *cds4-27* completely (Figure 5C), and could not biosynthesize the metabolite of ECO-0501 (Figure 5D). So, the pLYHMY87-5-I with dual sgRNAs and two homologous arms flanking the targeted cluster could improve the deletion efficiency in deleting larger fragment, which covers a size of 87.5 kb region in ECO-0501 BGC.

### 3.4 Improvement of vancomycin production through the deletion of ECO-0501 BGC

**FIGURE 6:**
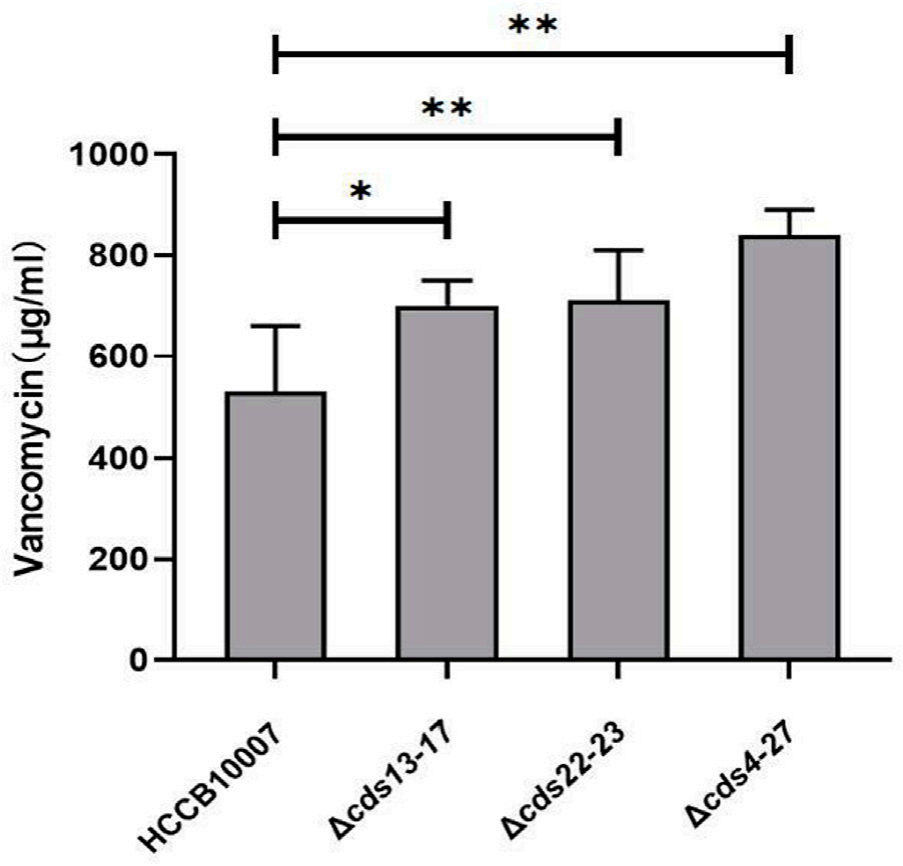
Improvement of vancomycin production. HPLC detection of vancomycin in the mutants indicated that the yield of vancomycin was increased owing to the successful deletions of gene clusters *cds13-17*, *cds22-23*, and *cds4-27* in ECO-0501 BGC. The position indicated by the red arrow was ECO-0501 peak. Original, original strain *A. keratiniphila* HCCB10007; Δ*eco-cds13-17*, the mutant *A. keratiniphila* HCCB10007 Δ*eco-cds13-17*; Δ*eco-cds22-23*, the mutant *A. keratiniphila* HCCB10007 Δ*eco-cds22-23*; Δ*eco-cds4-27*, the mutant *A. keratiniphila* HCCB10007 Δ*eco-cds4-27*. Error bars represented the standard deviations from three independent biological replicates of each strain. ^*^ *p* < 0.05, ^**^ *p* < 0.01.

*A. keratiniphila* HCCB10007 is well known for producing vancomycin and ECO-0501, and the both biosynthesis pathways share some common precursor (Shen et al., 2014; Xu et al., 2014). Because of the deletions of gene clusters *cds13-17*, *cds22-23* and *cds4-27* in the BGC of ECO-0501, the ability of vancomycin biosynthesis in the three mutants were all significantly improved, and the production of vancomycin increased by 30.54% ± 6.2%, 33.99% ± 8.6%, and 40.58% ± 7.5%, respectively (Figure 6).

## 4 Discussion

The CRISPR/Cas-mediated gene/genome editing system exhibits powerful efficiency and has been successfully applied in the actinomycetes *Streptomyces* species (Cobb et al., 2015; Wang et al., 2016). Both CRISPR/Cas9 and CRISPR/Cas12a-mediated gene editing systems have been reported deleting single gene (*rifZ*, *glnR* and *vdh*) and two genes *gtfDE* in the rare actinomycetes of *A. mediterranei* U32, *A. orientalis* AO-1 and *Amycolatopsis* sp. (Zhou et al., 2020; Qian et al., 2021; Zheng et al., 2021), however it is more desirable to develop a highly efficient CRISPR/Cas system to knock out large chromosomal fragments in *Amycolatopsis*.

All-in-one CRISPR/Cas9-mediated genome editing plasmid pKCcas9dO harbored the temperature-sensitive characteristic of replicon pSG5, apramycin-resistance gene (*aac3IV*), the thiostrepton-inducible promoter *tipA* and a codon optimized *cas9* from *S. pyogenes*, the promoter *J23119* and a target specific sgRNA, along with a pair of homologous recombination repair templates for HDR after DSB. It created the single gene deletions as well as whole antibiotic BGC deletions of up to 82.8 kb with an efficiency of 60%–100% in *S. coelicolor* M145 (Huang et al., 2015). It was also successfully applied in *S. pristinaespiralis* and *S. cinnamonensis* for deleting a 25.5 kb-long gene cluster and *dasR* with higher efficiency (Huang et al., 2015; Zhang et al., 2016). However, the gene editing plasmid pLYZYP01 derived from pKCcas9dO deleted the *vdh* gene with a lower efficiency of 10% in *Amycolatopsis* sp. (Zheng et al., 2021). Here, an efficient CRISPR/Cas9-mediated genome editing system, derived from pKCcas9dO, was reported in vancomycin-producing strain *A. keratiniphila* HCCB10007. Employing the established method, the deletions of large-size gene clusters were successfully achieved, and the production of vancomycin was increased 30%–40% by deleting BGCs of ECO-0501.

The critical issues to CRISPR/Cas9 systems are the toxicity of Cas9 in the specific strain and the poor expression of the *cas9* gene or that of sgRNA(s) (Alberti and Corre, 2019; Ding et al., 2020). Cas9/dCas9 has been demonstrated to be toxic in *Amycolatopsis* species, and no transformants were obtained when Cas9/dCas9 was expressed in *A. orientalis* AO-1 and *A. mediterranei* U32 (Zhou et al., 2020; Qian et al., 2021). In this study, the transformation efficiency of the plasmid pKCcas9dO was found much lower than that of the control plasmid. Therefore, the expressions of *scocas9* and sgRNA were needed to be tuned. It was required to confirm whether the *tipA* and J23119 promoters would be appropriate to drive the expressions of the *scocas9* and sgRNA of *A. keratiniphila*. Considering the susceptibility of the synthetic promoter J23119 to the surrounding sequence context (Jin et al., 2019), the strong constitutive promoter ermE*, which was proven to drive the overexpression of a type II thioesterase gene (*ECO-orf27*) to enhance the yield of ECO-0501 of *A. orientalis* dA9 (Shen et al., 2014), was used to transcribe target-specific sgRNA. However, the high-level expression of *scocas9* under control of *tipA* promoter still led to negative influence on the growth of the strain *A. keratiniphila* HCCB10007. In order to improve genome editing efficiency, the Cas9 protein was usually highly expressed by a strong promoter (Ding et al., 2020). However, the expression of *cas9* with strong promoter showed toxic effect to the host cells (Wang et al., 2016; Ding et al., 2020; Mitousis et al., 2020). To address this issue, one of strategies was to modulate *cas9* expression at the transcriptional levels (Ye et al., 2020; Zhao et al., 2020). Given that the stronger promoter *gapdh* drove the transcription of heterologous genes at high level in *Streptomyces* (Shao et al., 2013), and it was selected to drive the sgRNA expression of CRISPR/Cas9 plasmid pCRISPomyces-2 (Cobb et al., 2015; Wang et al., 2016), the endogenous *gapdh* promoter was attempted to lower the toxicity of Scocas9 to the cells of *A. keratiniphila* HCCB10007. As seen in the experiment, the transformation efficiency was improved. This result suggested that the Cas9 toxicity to the strain *A. keratiniphila* HCCB10007 somehow could be addressed by using endogenous *gapdh* promoter, as long as the expression level of Scocas9 was kept on the range of tolerance of the cells.

Furthermore, one dominant challenge is active, reliable and sufficient expression of Cas9 protein and sgRNA when CRISPR/Cas system is applied in non-model microorganisms (Ding et al., 2020). Generally, the strong promoters should be considered to guarantee sufficient Cas9 abundance for efficient CRISPR editing rate, and the strong expression of gRNA was also recommended for an efficient DNA target binding and CRISPR complex activation (Cobb et al., 2015; Huang et al., 2015; Ding et al., 2020). The CRISPR/Cas9 toolkits of pCRISPR-Cas9 and pCRISPomyces-2 were applied in *Streptomyces* successfully (Cobb et al., 2015; Tong et al., 2015; Wang et al., 2016), and their promoter combinations to drive the expression of *cas9* and sgRNA were *tipA*p/ermE*p, *rpsL*p (XC)/*gapdh*p (EL), respectively. The promoter *tipA* showed higher activity compared to ermE*p in *S. lividans* (Liu et al., 2016), while the strong promoters of *rpsL*p (XC) and *gapdh*p (EL) were confirmed similar activities in *S. lividans* (Shao et al., 2013). However, the promoter combination of the plasmid pCM4.4, which performed gene editing with high efficiency in *S. coelicolor,* was ermE*p/*gapdh*p (EL), and the activity of ermE*p for *cas9* expression was weaker than that of *gapdh*p (EL) for sgRNA expression (Ye et al., 2020). Thus, medium strength or weak promoters of *cas9* also showed high editing efficiency (Ding et al., 2020). The promoters which were selected to drive the expressions of CRISPR/Cas9 elements correlated with the strains. In *Amycolatopsis* sp. ATCC 39116, the Gene expression activated by *Km* ^*r*^p led to a six-fold lower glucuronidase activity in comparison to ermE*p (Fleige and Steinbüchel, 2014), and the deleting efficiency of *vdh* gene was only 10% with the promoter combination of *Km* ^*r*^p/ermE*p for driving the expressions of the *scocas9* and sgRNA (Zheng et al., 2021). In this study, with the help of the homologous arms and selected sgRNA, the promoter combination of *gapdh*p/ermE*p achieved a high-efficient deletion of large-size DNA fragments, but it is worth further studying the optimal promoters for driving CRISPR/Cas9 elements in *A. keratiniphila*.

Besides, the design of the sgRNA appeared however to influence considerably the efficiency of deletions (Tong et al., 2015; Alberti and Corre, 2019; Ding et al., 2020), and the GC content, binding site and sequence composition of sgRNA might influence on the editing efficiency (Tong et al., 2015; Ding et al., 2020; Liu et al., 2020; Zhang et al., 2020). But, it was not clearly observed in this study. So, more sgRNAs should be tested for high-efficient deletions of large fragments in *A. keratiniphila* HCCB10007.

It was proposed that the editing efficiency was somehow unrelated to the edited DNA size when knocking out several specifications of fragments ranging from 1.0–82.8 kb in *Streptomyces* (Huang et al., 2015). It seems possible to find an efficient sgRNA to achieve the purpose of knocking out a fragment of whatever size, but there was an upper limit of the edited DNA size with the same sgRNA in this study. When targeting large fragments of 12.7 kb and 21.2 kb, the plasmids could perform well and reach the high editing efficiency of more than 81%. However, when the targeting fragment became larger, such as 87.5 kb tried in this experiment, the editing plasmid did not work. In *S. lividans*, it was possible for excision of larger chromosomal segments of 31 kb *red* cluster by introducing a DSB at both ends and bridging the gap with a plasmid-borne editing template, and all four exconjugants displayed the edited genotype by using a dual-targeting pCRISPomyces-2 plasmid (Cobb et al., 2015). Similar results were observed that the deletion rates of the 31.6 kb *red* cluster and 52.9 kb *act/red* clusters rose up to 67% and 45% in *S. coelicolor* by dual-sgRNA strategy (Huang et al., 2015). In this study, the dual-sgRNA strategy could improve the deletion efficiency of CRISPR/Cas9 system in editing the larger DNA fragment of 87.5 kb. The simultaneous action of two sgRNAs located at both ends of 87.5 kb fragment might be the possible reason for the increase in efficiency. Although the dual sgRNA strategy was effective in larger gene fragment deletion, only modest editing efficiency was observed and the transformants greatly reduced. To improve the editing efficiency in *A. keratiniphila*, the optimization of the *cas9* codon and the extension of homologous arms lengths should be carefully considered in the case of larger DNA fragment deletion.

It is not unusual for actinomycetes that the two or more unrelated senondary metabolic pathways compete for common precursors, cofactors, energy sources, reducing power, etc., thus limiting the potential yield of the desired product (Baltz, 2016). The biosynthesis pathways of vancomycin and ECO-0501, belonging to the non-ribosomal peptide synthetase system and type I polyketide synthase system, respectively, share some common precursors, including malonyl-CoA and D-glucose (Banskota et al., 2006; Shen et al., 2014; Xu et al., 2014). malonyl-CoA is the initial precursor not only bond with the acyl carrier protein for the biosynthesis of the polyketide backbone of ECO-0501 (Banskota et al., 2006), but also catalyze to form 3,5-dihydroxyphenylglycine (L-Dpg), which involved in the heptapeptide backbone of vancomycin (Xu et al., 2014). Both vancomycin and ECO-0501 have the glycosyl groups which transformed from D-glucose (Banskota et al., 2006; Xu et al., 2014). Because of the relationship between the biosynthetic pathways of vancomycin and ECO-0501, the disrupted pathway of ECO-0501 would redirect the precursor flow into vancomycin biosynthetic pathway and lead to the higher level of vancomycin.

In conclusion, a highly efficient CRISPR/Cas9-mediated genome editing for large DNA fragment of ∼21 kb deletion was demonstrated in *A. keratiniphila* HCCB10007, and it accomplished larger DNA fragment of 87.5 kb deletion by dual sgRNA strategy. The improvement of vancomycin was realized by disrupting the competing secondary metabolic pathway of ECO-0501. This system would facilitate a wide variety of future studies in rare actinomycetes *Amycolatopsis* species, such as analysis of metabolic pathways, enhancement of secondary metabolites production, activation of silent BGCs, and industrial strain improvement.

## Data Availability

The original contributions presented in the study are included in the article/Supplementary Material, further inquiries can be directed to the corresponding author.
