# Supplementary material for: CRISPR/Cas9-mediated genome editing in vancomycin-producing strain Amycolatopsis keratiniphila
Source: Front Bioeng Biotechnol. 2023 Mar 3;11:1141176. doi: 10.3389/fbioe.2023.1141176 (PMC10020181; doi:10.3389/fbioe.2023.1141176)
Supplement: Supplementary file 1 [file Table1.DOCX]

Supplementary Material

CRISPR/Cas9-mediated genome editing in vancomycin-producing strain *Amycolatopsis keratiniphila*

Mengyi Hu^1^, Shuo Chen^1^, Yao Ni^1^, Wei Wei^2^, Wenwei Mao^1^, Mei Ge^2^, Xiuping Qian^1*^

*** Correspondence:** Corresponding Author: qianxp@sjtu.edu.cn

# Supplementary Table S1: Strains and plasmids

| **Strains/ Plasmids** | **Description** | **Sources** |
| --- | --- | --- |
| Strains |  |  |
| E. coli DH5α | *sμpE44 Δlacμ169(φ80lacZΔM15)*, used for plasmid cloning | Weidi |
| E. coli JM110 | *dam*^-^, *dcm*^-^, used for plasmid demethylation | Weidi |
| *A. keratiniphila* HCCB10007 | Vancomycin and ECO-0501-producing strain | [27, 28] |
| *A. keratiniphila* HCCB10007 *eGFP* Δ*gtfD* | *A. keratiniphila* HCCB10007 with insertion of *eGFP* and deletion of gtfD | This work |
| *A. keratiniphila* HCCB10007 Δ*eco-cds13-17* | *A. keratiniphila* HCCB10007 with deletion of CDS13-17 in ECO-0501 cluster | This work |
| *A. keratiniphila* HCCB10007 Δ*eco-cds22-23* | *A. keratiniphila* HCCB10007 with deletion of CDS22-23 in ECO-0501 cluster | This work |
| *A. keratiniphila* HCCB10007 Δ*eco-cds23* | *A. keratiniphila* HCCB10007 with deletion of CDS23 in ECO-0501 cluster | This work |
| *A. keratiniphila* HCCB10007 Δ*eco-cds4-27* | *A. keratiniphila* HCCB10007 with deletion of CDS4-27 in ECO-0501 cluster | This work |
| **Plasmids** |  |  |
| pMD19-T simple vector | Provide restriction sites | TaKaRa |
| pKC1139 | *acc(3)IV*, pSG5, pBR322, *oriT*_RK2_ | HonorGene |
| pKCCas9dO | *acc(3)IV*, pSG5, *tipA*-*Scocas9*, j23119, *actII-orf4* guide-RNA, homologous region flanking *act-orf4* | [30] |
| pLYZWG | *oriT*, *aac(3)IV*, *bla*, *Sce*I site, *eGFP*, *ermE*p* | [34] |
| pLYNY02 | pKCCas9dO with Cas9 under control of P*_tipA_* and *gtfD* sgRNA under control of P*_j23119_*, up/downstream homologous arms of *gtfD*, *eGFP* | This work |
| pLYNY03 | pKCCas9dO with Cas9 under control of P*_tipA_* and *gtfD* sgRNA under control of P*_ermE*_*, up/downstream homologous arms of *gtfD*, *eGFP* | This work |
| pLYNY04 | pKCCas9dO with Cas9 under control of P*gapdh* and *gtfD* sgRNA under control of P*_ermE*_*, up/downstream homologous arms of *gtfD*, *eGFP* | This work |
| pLYHMY7-1 | pLYNY04 with sgRNA 1, up/downstream homologous arms of CDS13-17 in ECO-0501 cluster | This work |
| pLYHMY7-2 | pLYNY04 with sgRNA 2, up/downstream homologous arms of CDS13-17 in ECO-0501 cluster | This work |
| pLYHMY7-3 | pLYNY04 with sgRNA 3, up/downstream homologous arms of CDS13-17 in ECO-0501 cluster | This work |
| pLYHMY7-4 | pLYNY04 with sgRNA 4, up/downstream homologous arms of CDS13-17 in ECO-0501 cluster | This work |
| pLYHMY7-5 | pLYNY04 with sgRNA 5, up/downstream homologous arms of CDS13-17 in ECO-0501 cluster | This work |
| pLYHMY7-6 | pLYNY04 with sgRNA 6, up/downstream homologous arms of CDS13-17 in ECO-0501 cluster | This work |
| pLYHMY21-I | pLYNY04 with sgRNA I, up/downstream homologous arms of CDS22-23 in ECO-0501 cluster | This work |
| pLYHMY21-II | pLYNY04 with sgRNA II, up/downstream homologous arms of CDS22-23 in ECO-0501 cluster | This work |
| pLYHMY21-III | pLYNY04 with sgRNA III, up/downstream homologous arms of CDS22-23 in ECO-0501 cluster | This work |
| pLYHMY12-I | pLYNY04 with sgRNA I, up/downstream homologous arms of CDS23 in ECO-0501 cluster | This work |
| pLYHMY87-I | pLYNY04 with sgRNA I, up/downstream homologous arms of CDS4-27 in ECO-0501 cluster | This work |
| pLYHMY87-5-I | pLYNY04 with sgRNA I and sgRNA 5, up/downstream homologous arms of CDS4-27 in ECO-0501 cluster | This work |

# Supplementary Table S2: Primers used in this study

| **Name** | **Sequence (5’→3’)** | **Purpose** |
| --- | --- | --- |
| **sgRNA** amplification | | |
| gRNADNrecom | acgttgtaaaacgacggccagtgccAAGCTTctcaaaaaaagcaccgac | *gtfD* specific sgRNA fragment |
| gtfDgRNAspc2 | tcagtcctaggtataatACTAGTcgtcgagatcgcggtgtcgcgttttagagctagaaa |  |
| gtfDgRNArecom | ggcacaatcgtgccggttggtaggaACTAGTcgtcgagatcgcggtgtcgcgttttagagctagaaa | *gtfD* sgRNA recombinant fragment |
| oligo-1F | atttctagctctaaaaccgtttgagccgggccaatttactagttcctaccaaccggcacg | sgRNA 1 |
| oligo-1R | cgtgccggttggtaggaactagtaaattggcccggctcaaacggttttagagctagaaat |  |
| oligo-2F | atttctagctctaaaactgttcctttgtgtacccaccactagttcctaccaaccggcacg | sgRNA 2 |
| oligo-2R | cgtgccggttggtaggaactagtggtgggtacacaaaggaacagttttagagctagaaat |  |
| oligo-3F | atttctagctctaaaaccaaaggacagaaaagaaaggactagttcctaccaaccggcacg | sgRNA 3 |
| oligo-3R | cgtgccggttggtaggaactagtcctttcttttctgtcctttggttttagagctagaaat |  |
| oligo-4F | atttctagctctaaaaccgaagcaccaacaacccctaactagttcctaccaaccggcacg | sgRNA 4 |
| oligo-4R | cgtgccggttggtaggaactagtTAGGGGTTGTTGGTGCTTCGgttttagagctagaaat |  |
| oligo-5F | atttctagctctaaaaccctttcttttctgtcctttgactagttcctaccaaccggcacg | sgRNA 5 |
| oligo-5R | cgtgccggttggtaggaactagtCAAAGGACAGAAAAGAAAGGgttttagagctagaaat |  |
| oligo-6F | atttctagctctaaaacacaggctctgagaggggagaactagttcctaccaaccggcacg | sgRNA 6 |
| oligo-6R | cgtgccggttggtaggaactagtTCTCCCCTCTCAGAGCCTGTgttttagagctagaaat |  |
| oligo-IF | atttctagctctaaaaccaagtcaggagatcccgagtactagttcctaccaaccggcacg | sgRNA I |
| oligo-IR | cgtgccggttggtaggaactagtactcgggatctcctgacttggttttagagctagaaat |  |
| oligo-IIF | atttctagctctaaaacccagaggagtgggccaccagactagttcctaccaaccggcacg | sgRNA II |
| oligo-IIR | cgtgccggttggtaggaactagtctggtggcccactcctctgggttttagagctagaaat |  |
| oligo-IIIF | atttctagctctaaaaccggatcttcacgaaacccggactagttcctaccaaccggcacg | sgRNA III |
| oligo-IIIIR | cgtgccggttggtaggaactagtccgggtttcgtgaagatccggttttagagctagaaat |  |
| **Homologous arm amplification** | | |
| Vcm8-F | AAGCTTagatcggtgagtcgctgctg | Upstream homologous arm of *gtfD* |
| Vcm8-R | tcacgtatttccccgCTGCAGGGTACCttcgctacccctgtttcgtg |  |
| Vcm10-F | aacaggggtagcgaaGGTACCCTGCAGcggggaaatacgtgatgcgt | Downstream homologous arm of *gtfD* |
| Vcm10-R | AAGCTTttggtgatgatcaggcggga |  |
| TYCZ-F | gtaaaacgacggccagtgccAAGCTTagatcggtgagtcgctgctg | Homologous arm of *gtfD* |
| TYCZ-R | gagtcggtgctttttttgagAAGCTTttggtgatgatcaggcggga |  |
| 13-17-arm-AF | acgacggccagtgccaagcttACGAGGACCACGATGACGA | Upstream homologous arm of CDS13-17 |
| 13-17-arm-AR | ttcgtctcgcatggtccgTTCGGGTGGACCGGGGAA |  |
| 13-17-arm-ZF | aaCGGACCATGCGAGACGAA | Downstream homologous arm of CDS13-17 |
| 13-17-arm-ZR | ggtgctttttttgagaagcttCTCGTCGGCGAACACGATC |  |
| 22-23-arm-AF | acgacggccagtgccaagcttGTGGAAGGCCGGGGACGACGT | Upstream homologous arm of CDS22-23 |
| 22-23-arm-AR | ggatccacgacCAGTATCAGGCGGGTCAGGA |  |
| 22-23-arm-ZF | ctgatactgGTCGTGGATCCCCGGCCT | Downstream homologous arm of CDS22-23 |
| 22-23-arm-ZR | ggtgctttttttgagaagcttAAGGGCGTGGTCAAGAACG |  |
| 23-arm-AF | gtaaaacgacggccagtgccGACTTGGAGCATCGTGCCGT | Upstream homologous arm of CDS23 |
| 23-arm-AR | gatccacgacCGCTTCGCGCACGTTCACCAG |  |
| 23-arm-ZF | gcgcgaagcgGTCGTGGATCCCCGGCCT | Downstream homologous arm of CDS23 |
| 23-arm-ZR | ggtgctttttttgagaagcttAAGGGCGTGGTCAAGAACG |  |
| 4-27-arm-AF | acgacggccagtgccaagcttCCGGATACACCAAGAGCACATCA | Upstream homologous arm of CDS4-27 |
| 4-27-arm-AR | acgggcgatcTTGCCGAGGAGCCTAGAGGAC |  |
| 4-27-arm-ZF | tcctcggcaaGATCGCCCGTCCCACCGAGCGT | Downstream homologous arm of CDS4-27 |
| 4-27-arm-ZR | ggtgctttttttgagaagcttGGATCAAGGCAACCTGCTGTG |  |
| **Others** |  | Amplification fragment |
| ermE-F | ggatttgttcagaacgctcggttgcTCTAGAgattctctagtatgcatgc | ermE* promoter |
| ermE-R | tcctaccaaccggcacgat |  |
| gapdh-F | acactcgcatgcatactagagaatcTCTAGAagctgagagttttcccaag | *gapdh* promoter |
| gapdh-R | ccaggccgatggagtacttcttgtcCATATGtgctgccactcccttgaga |  |
| sgRNA5-F | gcatgcatactagagaatctAAAAAAAGCACCGACTCG | sgRNA 5+ ermE* promoter |
| sgRNA5-R | ggaaaactctcagcttctagGATTCTCTAGTATGCATG |  |
| **Verify Primers** |  | **Target length (bp)** |
| Verify-g8-F | accggtcaaattgggcgtcg | 3340 |
| Verify-g8-R | gcagatgaacttcagggtca |  |
| Verify-10g-F | gtgcagtgcttcagccgcta | 3357 |
| Verify-10g-R | atccaccggccgtccgccgc |  |
| Verify-Gg-F | gagttcgaggcgcgctatcg | 1795/1969 |
| Verify-Gg-R | accggagcatgtcctcggcc |  |
| Verify-7in-F | atcctactgtcgggttcatcgg | 1491 |
| Verify-7in-R | ggtccacctttcttttctgtccttt |  |
| Verify-7out-F | ggggcacagcaacaaggaga | 6092 |
| Verify-7out-R | ggcataagtcggcaaatcgg |  |
| Verify-21in-F | tcgactgcgggatcgtgga | 1403 |
| Verify-21in-R | ggtgaggcgttctgcggac |  |
| Verify-21out-F | ccaagaccgaggaagcgttgc | 6602 |
| Verify-21out-R | cgtccgacctccatatcaccca |  |
| Verify-12in-F | gctgcacagcttcggtcctt | 1481 |
| Verify-12in-R | tcaccgagttcgaccgagag |  |
| Verify-12out-F | gacgccgaacaccttcatcg | 5169 |
| Verify-12out-R | atgtcggtgcgagcatcgtcct |  |
| Verify-87out-F | agtttcctttgacgccatgc | 6777 |
| Verify-87out-R | tggactctgtctggggtgtg |  |
